# The vulnerability of maintenance dialysis patients with COVID-19: mortality and risk factors from a developing country

**DOI:** 10.1080/07853890.2022.2075914

**Published:** 2022-05-20

**Authors:** Nabil Ahmed, Abdel Hadi Khderat, Alaa Sarsour, Ameed Taher, Ahmad Hammoudi, Zakaria Hamdan, Zaher Nazzal

**Affiliations:** a Registered Nurse, Kidney and Dialysis Section, An-Najah National University Hospital, Nablus, Palestine; b Jenin Government Hospital, Palestinian Ministry of Health, Jenin, Palestine; c Kidney and Dialysis Section, Department of Internal Medicine, An-Najah National University Hospital, Nablus, Palestine; d Department of Internal Medicine, Faculty of Medicine and Health Sciences, An-Najah National University, Nablus, Palestine; e Department of Family and Community Medicine, Faculty of Medicine and Health Sciences, An-Najah National University, Nablus, Palestine

**Keywords:** COVID-19, incidence, haemodialysis, peritoneal dialysis, mortality rate

## Abstract

Patients on maintenance dialysis therapy are especially vulnerable to COVID-19 and its complications. This study aimed to assess the incidence, epidemiological characteristics, and mortality rate of COVID-19 among maintenance dialysis patients. This retrospective observational chart review study included 548 patients from all dialysis units in the West Bank of Palestine who acquired COVID-19 between 5 March 2020, and 11 August 2021. We collected data on patients' demographics, clinical features, and outcomes. A multivariable logistic regression model was used to assess independent risk factors for COVID-19-related mortality. The incidence of COVID-19 among maintenance dialysis patients was 35.3%, as 548 out of 1554 patients have tested positive during the study period. Patients on haemodialysis were three times riskier to get infected than those on peritoneal dialysis (37% vs 11.3%). Half (50.2%) of infected patients required hospitalisation, and 24.5% were admitted to an intensive care unit, while the mortality rate stood at 26.8%. Old age, male sex, central venous catheter use, comorbid diabetes, smoking, and having an RH negative blood group type were determined to be significantly associated with increased risk of mortality. In conclusion, the incidence of COVID-19 among Palestinian maintenance dialysis patients was notably high, especially among haemodialysis patients. High rates of hospitalisation, ICU admission, intubation and death were observed, and predictive factors for COVID-19-related mortality were identified. Therefore, the implementation of strict infection control measures and promotion of home dialysis are warranted to reduce the infection rate.

**KEY MESSAGES**
- The incidence of COVID-19 among Palestinian maintenance dialysis patients was notably high; more than one-third of the total dialysis population acquired COVID-19, with haemodialysis patients being three times more likely to get infected compared to their peritoneal dialysis counterparts.
- The mortality rate among maintenance dialysis patients was 26.8%, more than 25 times higher than that of the general population. The risk of mortality was significantly increased with age, male sex, smoking, diabetes, and having central venous catheter as vascular access for haemodialysis.
- Strict infection control measures, as well as the promotion of home dialysis, are necessary to reduce the risk of infection.

## Introduction

Coronavirus disease 2019 (COVID-19) first appeared in Wuhan, China, in December 2019 [1], and was declared a pandemic by the World Health Organisation (WHO) on 11 March 2020 [2]. The clinical presentation of COVID-19 is unpredictable, ranging from asymptomatic cases to fatal ones. A large study conducted in China [3] showed that 81% of cases were mild to moderate, 14% were severe, and 5% were critical, leading to respiratory failure, shock and/or multi-organ system dysfunction [3]. The first case in Palestine was identified on 5 March 2020 at a hotel in Bethlehem [4], and by 1 December 2021, the number stands at 460,799 confirmed cases.

Patients undergoing maintenance dialysis are especially vulnerable to COVID-19 due to several factors. First of all, these patients need to go to the dialysis unit frequently for their treatment sessions, where many of them gather in a single space for several hours, making it impossible for them to fully self-isolate, leading to high infection rates among them [5]. They also tend to be older than the general population, and many of them have comorbid conditions [6], which puts them at a greater risk of developing a complicated course of illness. Furthermore, chronic kidney disease has been shown to be an independent risk factor for higher mortality among COVID-19 patients, probably due to the premature ageing of the immune system associated with uraemia [7]. Understanding the different factors that come into play in this group of patients is, thus, of particular importance.

The first study concerning the epidemiology of COVID-19 in maintenance haemodialysis patients came from China in March 2020 [8], and further studies have been published in different parts of the world ever since. However, most of these studies were single-centre and had limited sample sizes. They also tended to come from high and upper-middle-income countries, while data coming from less developed countries are still limited [9]. Therefore, we decided to conduct a large multi-centre study covering all the maintenance dialysis patients who have acquired COVID-19 in the West Bank (excluding Jerusalem) from March 2020 to August 2021. This study was intended to explore the incidence of COVID-19 infection, its epidemiological characteristics, prognostic predictors and mortality rates among the patients undergoing maintenance dialysis in Palestine.

## Material and methods

### Study design and subjects

This was a multi-centre retrospective observational chart review study. We included all end-stage renal disease (ESRD) patients on maintenance dialysis therapy at every dialysis unit in the West Bank (excluding Jerusalem due to access difficulties). Patients who were at least 18 years of age and had been dialysing for at least one month, whether they were receiving haemodialysis or peritoneal dialysis, were all included, given that they tested positive for COVID-19 during the period from 5 March 2020, to 11 August 2021. Those younger than 18 years of age and those who have been on dialysis therapy for less than one month were excluded. COVID-19 cases were identified based on the results of a polymerase chain reaction (PCR) of nasopharyngeal samples, following the Palestinian Ministry of Health's guidelines for COVID-19 diagnosis.

The study was approved by both An-Najah National University's Institutional Review Board (Ref #: Med. April 2021/12) and the Palestinian Ministry of Health's research committee. A waiver of informed consent was obtained since the study only used secondary data. In addition, we limited personally identifiable information to government-issued identification numbers and ensured that patients' identities were not disclosed to anyone outside the research team.

### Data collection

The researchers (NS, AK, and AS) personally reviewed the files of COVID-19-positive patients at every dialysis unit in the country and abstracted the relevant data. Collected data included patients' demographics (age, gender, and treatment location), characteristics (dialysis mode and duration, vascular access, previous comorbidities, tobacco use, and blood group), clinical presentation (fever, fatigue, dyspnoea, dry cough, sore throat, anosmia, myalgia, and gastrointestinal symptoms), and outcome (need for hospitalisation, need for ICU admission and/or intubation, and mortality), in addition to information about the COVID-19 diagnosis. Death was defined as any dialysis patient who died, and COVID-19 was listed as the underlying cause.

### Statistical analysis

Data processing and analysis were performed using the IBM SPSS Statistics for Windows, version 21 (IBM Corp., Armonk, NY, USA). We described categorical variables as frequency and percentages and continuous variables as median (interquartile range [IQR]). The mortality rate among study participants was computed with its 95% confidence interval. The Chi-squared test was used to compare categorical variables, while the Mann–Whitney U test was used to compare continuous variables. The significance level was set at a *p*-value of <.05. The Shapiro-Wilk test was used to determine if the distributions of continuous variables were normal. Based on the results of a univariable analysis, variables with a *p*-value of less than .10 were included in a multivariable logistic regression model to examine independent risk factors for COVID-19-related mortality. The receiver operating characteristic (ROC) curve and the area under the curve (AUC) were used to evaluate the accuracy of COVID-19-related symptoms in predicting mortality.

## Results

There were 1554 maintenance dialysis patients in the West Bank: 1448 were on haemodialysis (HD), representing 93.2% of the dialysis population, while 106 were on peritoneal dialysis (PD), representing 6.8% of the mentioned population. The number of those who acquired COVID-19 during the study period was 548, constituting 35.3% of the total dialysis population. However, the infection rate differed between the two groups; 37% of HD patients tested positive, in contrast to the only 11.3% seen in PD patients. Table 1 illustrates these findings.

**Table 1.** The rate of COVID-19 among dialysis patients in Palestine.

	Total number of dialysis patients	Infected with COVID-19	Rate (%)
Haemodialysis	1448	536	37.0
Female	550	233	42.4
Male	898	303	33.7
Peritoneal dialysis	106	12	11.3
Female	50	7	14.0
Male	56	5	8.9

### Demographics and clinical presentation

A total of 548 patients were included in our study. Patients' ages were in the range of 18–85 years, with a median of 60 years. There were 308 men (56.2%) and 240 women (43.8%), and almost a quarter were current smokers (23.4%). As for the dialysis duration, it ranged from 1 to 240 months, with a median of 29 months. The majority of the patients were on HD (97.8%), with PD patients being a small minority (2.2%). The current method of HD access was fistula in 366 (66.8%) patients.

Hypertension was the most common comorbidity, with nearly two-thirds (63.9%) of patients carrying the diagnosis. Diabetes came in a close second, as 59% had it. Other comorbidities followed: 37.6% had congestive heart failure (CHF), 25% had peripheral vascular disease (PVD), 8.8% had chronic obstructive pulmonary disease (COPD), and 2.7% had cancer. Table 2 shows the patients' demographics and characteristics.

**Table 2.** Patients' demographics and characteristics (*n* = 548).

	Frequency (%)	Median (range)
Age (years)		60 (18–85)
Gender		
Female	240 (43.8%)	
Male	308 (56.2%)	
Currently smoking	128 (23.4%)	
Health care setting		
Governmental	419 (76.5%)	
Non-governmental	129 (23.5%)	
Dialysis modality		
Haemodialysis	536 (97.8%)	
Peritoneal dialysis	12 (2.2%)	
Comorbidities		
Hypertension	350 (63.9%)	
Diabetes	323 (59.0%	
Heart failure	206 (37.6%)	
Peripheral vascular disease	137 (25.0%)	
COPD	48 (8.8%)	
Cancer	15 (2.7%)	
Dialysis access		
Fistula	366 (66.8%)	
Catheter	182 (33.2%)	
ABO blood group		
A	244 (45.4%)	
AB	48 (8.9%)	
B	69 (12.8%)	
O	176 (32.8%)	
Duration of dialysis (months)		29 (1–240)

**Figure 1.**
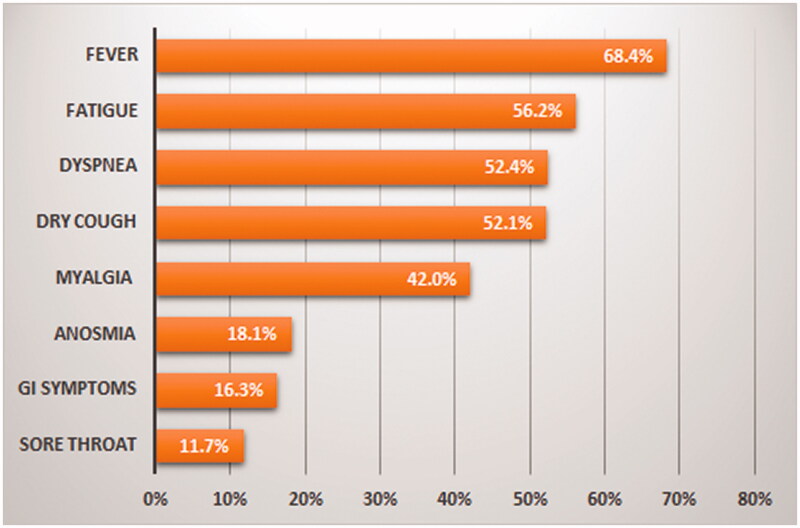
Frequency of COVID-19-related onset symptoms among dialysis patients.

Almost nine in ten (88.7%) patients reported at least one of the COVID-19-related symptoms, with fever being the most frequently reported (68.4%), followed by fatigue (56.2%), dyspnoea (52.4%), dry cough (52.1%), myalgia (42%), anosmia (18.1%), gastrointestinal symptoms (16.3%), and sore throat (11.7%). Nevertheless, 11.3% of patients had no symptoms. The frequency of COVID-19-related symptoms is shown in Figure 1.

### Clinical outcomes

Over half (50.2%) of the patients were hospitalised, and 48.7% of those were admitted to an intensive care unit (ICU), which means one in four (24.5%) patients were admitted to ICU. Moreover, 81 patients needed intubation; that is 29.5% of those hospitalised and 14.8% of the total study population. The length of stay in hospital varied from 1 to 120 days, with a median of 8 days. The length of ICU stay ranged from 1 to 90 days, with a median of 4 days. Finally, 147 patients died, putting the mortality rate at 26.8% (95% CI: 23.2–30.7%).

Comorbid diabetes and central venous catheter (CVC) use as vascular access were two variables significantly associated with increased rates of hospitalisation, ICU admission, and intubation (*p* value < .05). Old age was associated with higher rates of both ICU admission and intubation as well (*p* value < .05). Furthermore, male sex and comorbid CHF, COPD, and hypertension were all associated with increased risk of intubation (*p* value < .05). (Table 3)

**Table 3.** Hospitalisation, ICU admission, and intubation with patients' background and clinical characteristics.

	Hospitalised		Admitted to ICU		Intubated	
	*n* (rate)	*p*-Value*	*n* (rate)	*p*-Value*	*n* (rate)	*p*-Value*
Age (years)		.119**		.015**		<.001**
Yes	61 (22–103)		62.5 (22–90)		66 (22–88)	
No	59 (18–85)		59 (22–103)		59 (18–103)	
Gender		.338		.073		.003
Female	126 (52.5%)		54 (42.9%)		24 (10.0%)	
Male	149 (48.4%)		80 (53.7%)		59 (19.2%)	
Smoking		.576		.346		.194
Yes	67 (52.3%)		36 (53.7%)		24 (18.8%)	
No	208 (49.5%)		98 (47.1 %)		59 (14.0%)	
ABO blood group		.759		.489		.778
A	119 (48.8%)		52 (43.7%)		31 (12.7%)	
AB	22 (45.8%)		11 (50.0%)		8 (16.7%)	
B	38 (55.1%)		18 (47.4%)		10 (14.5%)	
O	88 (50.0%)		48 (54.5%)		28 (15.9%)	
Rh blood group		.429		.427		.677
Positive	240 (49.2%)		114 (47.5%)		69 (14.1%)	
Negative	27 (55.1%)		15 (55.6%)		8 (16.3%)	
Health care setting		.001		.143		.897
Governmental	227 (54.2%)		106 (46.7%)		63 (15.0%)	
Non-governmental	48 (37.2%)		28 (58.3%)		20 (15.5%)	
Dialysis modality		.990		.086		.882
Haemodialysis	269 (50.2%)		129 (48.0%)		81 (15.1%)	
Peritoneal dialysis	06 (50.0%)		5 (83.3%)		2 (16.7%)	
Dialysis access		.047		.043		.002
Fistula	137 (47.3%)		75 (43.4%)		43 (11.7%)	
Catheter	96 (56.5%)		54 (56.3%)		40 (22.0%)	
Diabetes		.003		.008		.002
Yes	179 (55.4%)		98 (54.7%)		62 (19.2%)	
No	95 (42.4%)		36 (37.9%)		21 (9.3%)	
Hypertension		.065		.260		.048
Yes	186 (53.1%)		95 (51.1%)		61 (17.4%)	
No	89 (44.9%)		39 (43.8%)		22 (11.1%)	
CHF		.039		.089		.008
Yes	115 (55.8%)		63 (54.8%)		42 (20.4%)	
No	160 (46.8%)		71 (44.4%)		41 (12.0%)	
COPD		.037		.270		.046
Yes	31 (64.6%)		18 (58.1%)		12 (25.0%)	
No	244 (48.8%)		116 (47.5%)		71 (14.2%)	
PVD		.068		.283		.630
Yes	78 (56.9%)		34 (43.6%)		19 (13.9%)	
No	197 (47.9%)		100 (50.8%)		64 (15.6%	
Dialysis duration (months)		.730**		.730**		.998**
Yes	30 (1–240)		30 (2–216)		34 (1–240)	
No	29 (1–144)		29 (1–144)		29 (1–216)	

*Chi-squared test; **Mann–Whitney U test.

### Predictors of mortality

The univariable and multivariable analyses of the factors associated with COVID-19-related mortality among maintenance dialysis patients are presented in Table 4. Age, gender, smoking status, Rh blood group, type of dialysis access, diabetes, and CHF were all associated with mortality using univariable analysis. The multivariable analysis showed a statistically significant increase in mortality as age increased (aOR: 1.1, 95%CI: 1.06–1.19). Additionally, men (aOR: 1.8, 95%CI: 1.1–2.7), smokers (aOR: 2.3, 95%CI: 1.4–3.8), patients with negative Rh blood group types (aOR: 2.2, 95%CI: 1.1–4.0), patients using CVC for dialysis (aOR: 2.2, 95%CI: 1.5–3.4), and those with diabetes (aOR: 2.2, 95%CI: 1.4–3.5) were all found to have a significantly higher mortality risk according to multivariable analysis.

**Table 4.** Univariable and multivariable analyses of COVID-19-related mortality predictors among maintenance dialysis patients.

	COVID-19 mortality			Multivariable analysis	
	Yes	No	*p*-Value*	*a*OR (95% CI*)*	*ap-*Value
Age in years median (range)	66 (22–103)	58 (18–85)	<.001**	1.1 (1.05–1.16)	<.001
Gender					
Female	50 (20.8%)	190 (79.2%)	.005	1.8 (1.1–2.7)	.029
Male^†^	97 (31.5%)	211 (68.5%)			
Smoking					
Yes	49 (38.3%)	79 (61.7%)	.001	2.3 (1.4–3.7)	.001
No^†^	98 (23.3%)	322 (76.7%)			
ABO Blood groups					
A	64 (26.2%)	180 (73.8%)			
AB	12 (25.0%)	36 (75.0%)	.951	–	–
B	20 (29.0%)	49 (71.0%)			
O	45 (25.6%)	131 (74.4%)			
Rh blood group					
Positive	122 (25.0%)	366 (75.0%)	.037	2.2 (1.1–4.0)	.040
Negative	19 (38.8%)	30 (61.2%)			
Health care setting					
Governmental	118 (28.2%)	301 (71.8%)	.203	–	–
Non-governmental	29 (22.5%)	100 (77.5%)			
Dialysis modality					
Haemodialysis	143 (26.7%)	393 (73.3%)	.608	–	–
Peritoneal dialysis	4 (33.3%)	8 (66.7%)			
Dialysis access					
AVF^†^	77 (21.0%)	289 (79.0%)	<.001	2.2 (1.4–3.7)	<.001
Catheter	66 (38.8%)	112 (61.5%)			
Diabetes					
Yes	114 (35.3%)	209 (64.7%)	<.001	2.3 (1.4–3.6)	.001
No^†^	33 (14.7%)	192 (85.3%)			
Hypertension					
Yes	98 (28.0%)	252 (72.0%)	.409	–	–
No	49 (24.7%)	149 (75.3%)			
CHF					
Yes	67 (32.5%)	139 (67.5%)	.019	1.1 (.668–1.6)	.873
No	80 (23.4%)	262 (76.6%)			
COPD					
Yes	21 (43.8%)	27 (56.2%)	.062	2.0 (0.968–4.0)	.056
No^†^	126 (25.2%)	374 (74.8%)			
PVD					
Yes	36 (26.3%)	101 (73.7%)	.867	–	–
No	111 (27.0%)	300 (73.0%)			
Dialysis duration in months median (range)	29 (1–240)	30 (1–216)	.256**		

†: reference group, *Chi-squared test, **Mann–Whitney U test, aOR: adjusted odds ratio, CI: Confidence Interval, a*p*-value: adjusted *p*-value.

**Figure 2.**
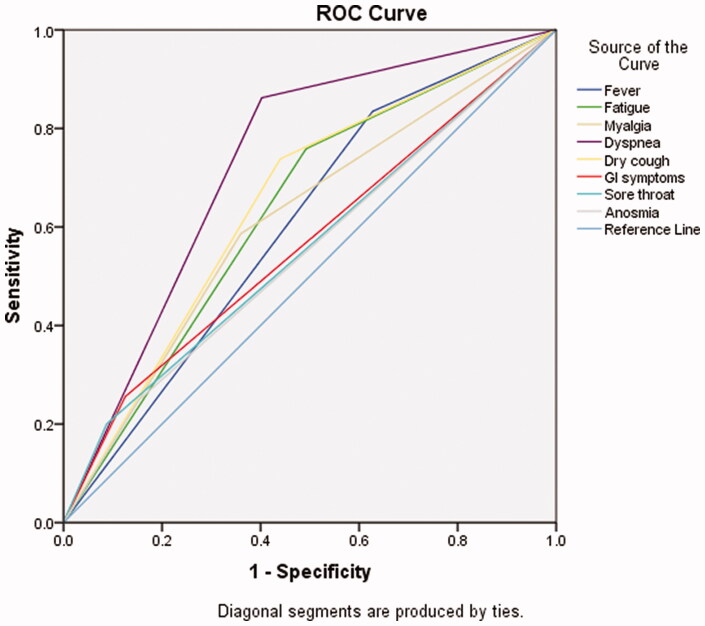
ROC curve for COVID-19-related symptoms in predicting mortality.

Furthermore, the accuracy of COVID-19-related symptoms in predicting mortality was assessed using the ROC curve. All COVID-19-related symptoms were associated with increased rates of hospitalisation and mortality. However, dyspnoea and dry cough specifically were the most powerful predictors of mortality among infected dialysis patients, as indicated by the AUC (Figure 2). The AUC was calculated as 0.73 (0.69–0.78) for dyspnoea and 0.65 (0.60–0.70) for dry cough.

## Discussion

This large multi-centre study has illustrated the incidence, clinical course, and outcome of COVID-19 infection among the patients undergoing maintenance dialysis in the West Bank. Data coming from low to lower-middle-income countries concerning this topic have been lacking [9]. To the best of our knowledge, our study is the first of its kind coming from a lower-middle-income country. It is also one of the only very few studies done at a national level [10,11].

Out of 1554 maintenance dialysis patients in the West Bank, 548 acquired COVID-19 during the study period, amounting to 35.3% of the total dialysis population. This rate is much higher than other studies; 7.1% in Qatar [11] and 12.1% in the United States [10], for example. This discrepancy might be explained by the variation of the periods through which different studies were conducted; five months in the Qatari study, ten months in the American study and 17 months in ours. However, limited resources and suboptimal adherence to infection control precautions could have also played a role.

Our results also showed that HD patients were more than three times more likely to contract COVID-19 compared to their PD counterparts (37% vs 11.3%). This is not surprising given that self-isolation is harder to achieve for HD patients, as many of them are bound to gather in limited and closed spaces for a considerable amount of time for their treatment sessions, usually for three times a week. Many of them also have to be in the close presence of others during transportation to and from dialysis centres. This finding is also consistent with what has been found in previous studies [12,13]. Additionally, these findings have prompted leading global kidney disease organisations to advocate for increased access and uptake of PD to minimise infection rates among dialysis patients, an especially vulnerable group to worse outcomes [14]. This would also hold true in the event of any pandemic that might hit in the future.

Out of 1554 maintenance dialysis patients in the West Bank, only 106 of them used PD, representing just 6.8% of the dialysis patient population, well below the estimated global average of 11% [15]. A good number of studies have examined the reasons behind the low appeal of PD in the eyes of both physicians and patients, but no such study has been done in Palestine. Conducting one, therefore, can be valuable.

Moreover, our study revealed that the mortality rate among maintenance dialysis patients in Palestine stood at 26.8%, more than 25 times higher than that of the general population [16]. However, it was within the range of mortality rates reported from other studies of comparable sample sizes: 16.2% in Turkey [17], 18.8% in Brazil [18], 24.9% in the United States [19], and 30.8% in Japan [20]. This variation might, at least in part, be explained by the difference in their patients' median age. However, it is worth noting that the median age of the American and the Turkish studies was higher than ours (67, 63 and 60 respectively), despite our mortality rate is higher than theirs. The Brazilian study didn't report their median age. A combination of a high prevalence of diabetes and limited medical resources might explain why our median age is younger than that found in other countries.

The risk of mortality was significantly increased with age and smoking, in accordance with the results of previous studies [20,21]. We also discovered that men had a higher mortality risk, which is consistent with the findings of a huge study done at the national level in the United States [10], even though this association was not evident in many other studies [17,18].

Having CVC as vascular access for haemodialysis represented yet another solid independent risk factor, also in accordance with the results of previous studies [18,22]. CVC access has been shown to be an independent risk of mortality and morbidity in multiple studies. In a large meta-analysis of 875,269 vascular accesses, mortality at two years was the highest among other types of vascular access; grafts and fistulas; 26%, 17%, and 15%, respectively [23]. Another large meta-analysis showed a higher risk of all-cause mortality, fatal infections, and cardiovascular events compared to patients using fistulas [24]. Data published on mortality and vascular access in Palestine also revealed that central venous dialysis catheters have a two-fold increase in mortality compared to arteriovenous fistulas [25]. The published literature demonstrates that AVFs are associated with improved outcomes when compared to CVC, and it is not surprising that similar findings were observed with COVID-19. Murt et al. demonstrated in their study that using AFVs as vascular access improves the survival of COVID-19-infected haemodialysis patients [26]. The increase in mortality in CVC patients may be due to the higher risk of central line bloodstream infections, as we don't have the precise data on the clinical course of patients who died.

Furthermore, our results showed that diabetes and congestive heart failure increased the risk of death, but only diabetes remained an independent risk factor after multivariable analysis. The prevalence of diabetes is relatively high in Palestine, affecting 15.3% of the population in 2010 and an estimated 20.8% in 2020 [27], putting it on par with countries with the highest diabetes prevalence in the world. Our data has also demonstrated this, as 59% of patients carried the diagnosis. Recognising their vulnerability early on is, therefore, crucial. However, it is worth mentioning that some studies have failed to demonstrate diabetes as an independent risk factor for mortality in the dialysis population. This might be due to the small sample size in some of them [6], and it might be due to the differences in the level of glycemic control among different patient populations in others. Indeed, it could very well be the case that uncontrolled blood sugar level is linked to worse COVID-19 outcomes, as has been suggested previously [28].

Data concerning the association between the other comorbidities and mortality have been conflicting; CHF, COPD, and PVD have all been implicated by some studies but not by others [17,20,29]. No such association has been established in our results after using multivariable analysis.

A unique finding in our results was the effect of the RH blood group type. The mortality rate stood at 38.8% in those with RH negative blood groups, but only 25% of those with RH positive blood groups died. The association between the lack of the RH antigen and mortality risk remained significant after using both univariable and multivariable analyses. Further studies are needed to confirm such association. However, our results did not demonstrate any relationship between ABO blood group types and clinical outcome, despite some studies claiming otherwise [30,31].

Patients who presented with symptoms were more likely to develop a complicated course of illness and had a greater mortality risk compared to those who were asymptomatic, as one would expect. That included all the symptoms we investigated: fever, fatigue, myalgia, dyspnoea, dry cough, sore throat, anosmia and gastrointestinal symptoms. However, dyspnoea and dry cough were the most significant predictors of mortality. Some studies have found such association with only some of these symptoms [17], but this could be due to the small sample size. Moreover, 11.3% of our patients were asymptomatic, similar to the percentages found in previous studies [17,18].

This study was not without limitations. Firstly, the lack of a universal screening protocol adopted by all the dialysis centres meant that some COVID-19 cases might have gone unnoticed. So the rate of infection could have been even higher. Secondly, patient data were obtained from medical records retrospectively and indirectly, which limited our ability to gather further information that we felt might have been of value. Additionally, we could not collect patients' laboratory parameters and include them in our study, which would have yielded further helpful information. Lastly, vaccines were not available in Palestine at the time of the study, limiting the ability to study their impact; this point could be the subject of a future study. Nevertheless, this study was one of only a few of its kind done at a national level. It was also the first coming from a lower-middle-income country with relatively large sample size and a multi-centre nature, offering a new and needed perspective.

## Conclusion

In conclusion, the incidence of COVID-19 among Palestinian maintenance dialysis patients was notably high, with HD patients being three times more likely to get infected compared to their PD counterparts. Implementation of strict infection control measures and promotion of home dialysis is, thus, warranted to reduce the infection rate. The study also demonstrated a strikingly high mortality rate among infected dialysis patients. Old age, male sex, CVC use, diabetes, smoking, and RH negative blood group type were determined as the factors associated with increased mortality risk in these patients.

## Data Availability

The authors confirm that the data supporting the findings of this study are available within the article and its supplementary materials.
